# Diagnostic Accuracy of Pulp Sensibility Tests and Radiographic Findings for Endodontic-Periodontal Lesions

**DOI:** 10.7759/cureus.111365

**Published:** 2026-06-23

**Authors:** Sameer Chauhan, Smita D Dutta, G. Suryamohan, Shanila R Suravarapu, A. Vinod Kumar, Surabhi Mahawar

**Affiliations:** 1 Department of Prosthodontics, K.M. Shah Dental College and Hospital, Sumandeep Vidyapeeth (Deemed to be University), Vadodara, IND; 2 Department of Conservative Dental Sciences and Endodontics, College of Dentistry, Qassim University, Buraydah, SAU; 3 Department of Conservative Dentistry and Endodontics, Lenora Institute of Dental Sciences, Rajahmundry, IND; 4 Department of Conservative Dentistry and Endodontics, Meghna Institute of Dental Sciences, Nizamabad, IND; 5 Department of Oral and Maxillofacial Surgery, Ram Krishna Dharmarth Foundation (RKDF) Dental College and Research Centre, Bhopal, IND

**Keywords:** cone-beam computed tomography, diagnostic imaging, endodontics, periodontitis, pulp vitality test

## Abstract

Introduction: Accurate diagnosis of endodontic-periodontal (endo-perio) lesions remains challenging because pulpal and periodontal diseases often present with overlapping clinical and radiographic features. This study aimed to evaluate and compare the diagnostic accuracy of pulp sensibility tests and radiographic findings for endo-perio lesions using latent class analysis.

Materials and methods: This prospective cross-sectional diagnostic accuracy study included 100 teeth diagnosed with endo-perio lesions. Clinical examination, periodontal assessment, pulp sensibility testing, and radiographic evaluation were performed for all included teeth. Pulp sensibility assessment included cold testing using Endo-Ice and electric pulp testing. Digital periapical radiographs and cone-beam computed tomography were used for radiographic evaluation in selected cases. Lesions were categorized as endo-origin, perio-origin, or true combined lesions. Latent class analysis was used to estimate diagnostic sensitivity, specificity, predictive values, and prevalence in the absence of a definitive gold standard. Agreement between pulp sensibility tests and radiographic findings was analysed using Cohen’s kappa statistics.

Results: Among the evaluated teeth, endo-origin lesions were identified in 42 (42.0%), perio-origin lesions in 34 (34.0%), and true combined lesions in 24 (24.0%). Most endo-origin lesions demonstrated non-vital pulp responses, which were observed in 32 teeth (76.2%), whereas normal pulp responses were predominantly associated with perio-origin lesions, which were identified in 18 teeth (52.9%). Latent class analysis demonstrated an acceptable model fit (p = 0.34). Pulp sensibility tests showed significantly higher sensitivity than radiographic evaluation. Radiographic findings demonstrated a higher estimated specificity (85%; 95% CI: 76-94%) than pulp sensibility tests (78%; 95% CI: 68-88%). Agreement analysis showed a fair-to-moderate agreement between the two diagnostic methods.

Conclusion: Pulp sensibility tests demonstrated superior sensitivity, whereas radiographic findings showed comparatively greater specificity for diagnosing endo-perio lesions. A combined diagnostic approach using both sensibility testing and radiographic assessment may improve diagnostic accuracy and support effective treatment planning.

## Introduction

Endodontic-periodontal (endo-perio) lesions represent a challenging clinical condition in dental practice because of the close anatomical and pathological relationship between the pulpal and periodontal tissues. The communication pathways between these tissues, including dentinal tubules, accessory canals, and apical foramen, permit the spread of infection from one tissue to another, resulting in lesions with overlapping clinical and radiographic features [1]. Accurate identification of the primary source of infection is essential because the treatment approach, prognosis, and long-term survival of the tooth depend largely on whether the lesion is primarily endodontic, periodontal, or a true combined lesion [2].

Diagnosis of endo-perio lesions commonly relies on a combination of clinical examination, pulp sensibility testing, periodontal assessment, and radiographic evaluation [3]. Pulp sensibility tests (PSTs), such as cold and electric pulp testing, are routinely used to determine the sensory status of pulp [4]. These tests are simple, inexpensive, and widely accepted in clinical practice; however, they evaluate neural responses rather than true vascular vitality and may therefore produce inaccurate results under certain pathological conditions. False-positive and false-negative responses may occur in teeth with extensive periodontal involvement, calcified canals, a history of trauma, or inflammatory changes [5].

Radiographic examination also plays a major role in the diagnosis of endo-perio lesions by identifying periapical radiolucencies, furcation involvement, marginal bone loss, and periodontal ligament widening [6]. Conventional periapical radiographs and cone beam computed tomography (CBCT) provide valuable structural information regarding the extent and pattern of tissue destruction [3,6]. Nevertheless, radiographic interpretation alone may not always reliably differentiate between endodontic and periodontal lesions, because similar radiographic appearances can occur in both conditions.

Although both PSTs and radiographic findings are routinely used for clinical diagnosis, evidence comparing their diagnostic performance in endo-perio lesions remains limited. In addition, the absence of a definitive gold standard for determining the true disease status makes it difficult to assess the accuracy of these diagnostic methods. Latent class analysis (LCA) has recently gained importance as a statistical approach for evaluating diagnostic tests when no perfect reference standard exists [7].

Therefore, this study aimed to evaluate and compare the diagnostic accuracy of PSTs and radiographic findings in the diagnosis of endo-perio lesions using LCA. The primary objective of this study was to evaluate the diagnostic accuracy of pulp sensibility testing and radiographic assessment for identifying endodontic/non-vital and periodontal/vital disease states using LCA. The secondary objectives were to assess the agreement between pulp sensibility testing and radiographic classifications and to examine the distribution of lesion origin categories in relation to clinical and diagnostic findings.

## Materials and methods

Study design

This prospective cross-sectional diagnostic accuracy study was conducted in the Department of Conservative Dentistry and Endodontics at the Lenora Institute of Dental Sciences, Rajahmundry, India, from April 2022 to August 2023. The study protocol adhered to the Strengthening the Reporting of Observational Studies in Epidemiology (STROBE) guidelines for cross-sectional studies [8]. Ethical approval for this study was obtained from the Institutional Ethics Committee of Lenora Institute of Dental Sciences (Approval No. 52/IEC/LIDS/2022). This study was conducted in accordance with the ethical principles outlined in the Declaration of Helsinki. All participants were informed about the purpose, methodology, benefits, and possible risks associated with the study, and written informed consent was obtained prior to enrolment.

Eligibility criteria and participant selection

Participants were selected based on predefined inclusion and exclusion criteria to ensure diagnostic accuracy and standardization of the study sample (Table 1).

**Table 1:** Inclusion and exclusion criteria for participant selection. PPD: probing pocket depth.

Inclusion criteria	Exclusion criteria
Systemically healthy adult patients aged ≥18 years	Teeth with full-coverage restorations or crowns preventing reliable pulp testing
Patients presenting with at least one permanent tooth clinically diagnosed with an endo-perio lesion	Teeth with previous endodontic treatment
Teeth diagnosed with primary endodontic lesions with secondary periodontal involvement	Teeth with intracanal posts
Teeth diagnosed with primary periodontal lesions with secondary endodontic involvement	Teeth with immature apices
Teeth diagnosed with true combined endo-perio lesions	Teeth with root fractures
Teeth with PPD ≥5 mm	Teeth with external resorption
Teeth with attachment loss extending to the apical third of the root surface in at least one site	Patients with recent trauma to the involved tooth within the previous six weeks
Radiographic evidence of bone loss involving the periapical, furcation, or marginal regions	Pregnant or lactating women
Patients willing to undergo pulp sensibility testing and radiographic examination	Patients receiving analgesics, sedatives, opioids, gabapentinoids, or medications affecting pain perception
Only one tooth per participant included to ensure independence of observations	Individuals with cognitive impairment or inability to respond reliably to vitality testing

Sample size estimation

The sample size was calculated based on a previous study that reported the sensitivity and specificity of PSTs for the detection of pulpal necrosis [9]. Expected sensitivity and specificity values of 75% and 85%, respectively, were used for the calculation. Using G*Power software (version 3.1.9; Heinrich Heine University, Düsseldorf, Germany), with a power of 80%, a two-sided alpha level of 0.05, and an anticipated clinically meaningful difference of 20% in diagnostic accuracy between the evaluated modalities, the minimum required sample size was estimated to be 82 teeth. Considering the possibility of incomplete data acquisition, inconclusive test responses, and other non-evaluable measurements, the target sample size was increased by approximately 15%, resulting in a final sample size of 100 teeth. Although the LCA evaluated a broader latent disease state representing endodontic/non-vital versus periodontal/vital involvement, pulpal status constituted a key component of the latent construct. Therefore, published diagnostic accuracy estimates for pulpal necrosis were considered the most appropriate parameters for sample size determination. The sample size calculation was intended primarily to support the estimation and comparison of diagnostic accuracy measures, particularly sensitivity and specificity, rather than agreement statistics.

Clinical examination and methodology

Before study initiation, the examiner underwent calibration and training sessions for periodontal probing, mobility assessment, and clinical evaluation of endo-perio lesions using a standardized examination protocol. Calibration was performed through repeated assessments of a set of representative cases under faculty supervision until consistent measurements and diagnostic interpretations were achieved. All clinical examinations during the study were subsequently performed by the same calibrated examiner to ensure uniformity of data collection. The examination included visual inspection, periodontal probing, mobility assessment, and evaluation of pulpal status. Probing pocket depth (PPD) measurements were recorded at six sites per tooth using a University of North Carolina-15 periodontal probe (Hu-Friedy Manufacturing Co., LLC, Chicago, Illinois, USA). Tooth mobility was assessed clinically and graded according to the Miller classification.

To minimize order bias, the sequence of pulp sensibility testing and radiographic assessment was randomized for each participant using a computer-generated random allocation sequence prepared before data collection. Participants were assigned to one of two assessment orders: pulp sensibility testing followed by radiographic evaluation, or radiographic evaluation followed by pulp sensibility testing. All PSTs were performed under standardized clinical conditions. The cold test was first conducted using an Endo-Ice refrigerant spray containing 1,1,1,2-tetrafluoroethane (Coltene Whaledent Inc., Cuyahoga Falls, Ohio, USA) [10]. The refrigerant was sprayed onto a sterile cotton pellet, which was then applied to the middle third of the buccal surface of the tooth after isolation and drying. Patients' responses were recorded as normal, hyperresponsive, hyporesponsive, or negative, depending on the intensity and duration of the sensation experienced. A normal response was defined as a sharp transient pain lasting less than two seconds, whereas a lingering response exceeding 10 seconds was categorized as hyperresponsive. A delayed or dull sensation was considered hyporesponsive, and the absence of any sensation was categorized as a negative response [10].

Following a washout period of five minutes, electric pulp testing was performed using a Pulp Tester (Analytic Technology Corporation, Redmond, Washington, USA) [5]. A five-minute interval was selected to allow dissipation of transient sensory effects induced by cold stimulation before electric pulp testing. Where feasible, responses were interpreted relative to a clinically sound reference tooth, preferably the contralateral homologous tooth or, when unavailable, an adjacent sound tooth. The tooth surface was cleaned and dried before application of a conducting medium. The electrode was placed on the middle third of the buccal surface, and the numerical value corresponding to the patient’s first sensation was recorded. Each test was repeated twice, and the mean value was considered for analysis. Teeth demonstrating response patterns comparable to the reference tooth were categorized as vital, whereas those exhibiting delayed responses were categorized as having reduced vitality. Teeth showing no response at the maximum stimulation level were considered non-vital [5]. Composite PST outcomes were subsequently determined. Teeth showing negative responses to both cold and electric pulp testing were classified as non-vital, whereas those showing discordant responses were documented separately.

Radiographic evaluation was performed using digital intraoral periapical radiographs obtained with the paralleling technique using an X-ray unit operating at 70 kVp and 8 mA with an exposure time of 0.2 seconds. In cases where anatomical overlap limited visualization, CBCT scans were acquired with a limited field of view of 5 × 5 cm and a voxel size of 0.125 mm. Detailed acquisition parameters, including unit model, tube voltage, tube current, and exposure time, were not available in the archived imaging records. Such additional imaging was required in only three cases. All radiographs and CBCT scans were obtained by a single trained radiographer to ensure standardization.

Radiographic interpretation was independently performed by two blinded oral and maxillofacial radiologists after calibration. Agreement in the assessment of radiographic features, including the presence and extent of periapical radiolucency, furcation involvement, marginal bone loss pattern, periodontal ligament space widening, and evidence of root fractures or external root resorption, demonstrated excellent consistency (ICC > 0.85). Lesions were subsequently classified radiographically as predominant endodontic, predominant periodontal, or true combined based on the pattern and location of bone destruction. Radiographic classification was based exclusively on imaging findings. Predominant endodontic lesions were characterized by periapical radiolucencies centered on the root apex with minimal marginal bone loss; predominant periodontal lesions demonstrated marginal or furcation bone loss extending coronally to apically without a primary periapical lesion; and true combined lesions exhibited concurrent periapical and periodontal bone destruction with radiographic communication between the lesions. Clinical periodontal findings were not used in radiographic classification.

All findings were documented using coded case report forms. The collected data were entered into a password-protected electronic database with double data entry verification to minimize transcription errors. The clinician performing the pulp vitality testing was blinded to the radiographic findings, whereas the radiologists were blinded to the PST outcomes and clinical periodontal findings.

To minimize factors known to influence PST responses, a standardized testing protocol was employed, including exclusion of patients with recent dental trauma or medications affecting pain perception, tooth isolation and drying before testing, repeated measurements, and interpretation relative to a control tooth where applicable. To minimize observer bias, radiographic assessments were performed independently by two calibrated and blinded oral and maxillofacial radiologists using predefined diagnostic criteria, including periapical radiolucency, furcation involvement, marginal bone loss pattern, and periodontal ligament space widening.

Statistical analysis

LCA and bootstrap resampling were performed using R version 4.4.1 (R Foundation for Statistical Computing, Vienna, Austria) during the data analysis stage following completion of participant recruitment. A two-sided p-value of less than 0.05 was considered statistically significant. As no definitive gold standard exists for diagnosing endo-perio lesions, LCA was used as the primary statistical approach to estimate the diagnostic accuracy of the pulp vitality tests and radiographic findings. A binary latent variable representing the true disease state was constructed to classify lesions as endodontic/non-vital or periodontal/vital. The latent class model included two observed diagnostic indicators: the composite PST outcome and radiographic assessment findings. No external reference standard or clinician-assigned lesion category was used to define the latent classes. The clinical classifications of endo-origin, perio-origin, and true combined lesions were used only for descriptive reporting and were not incorporated into the latent class model. For the analysis, discordant cold test and electric pulp test responses were classified as reduced vitality and retained within the composite PST indicator.

Sensitivity, specificity, and prevalence estimates were calculated with bootstrap-derived 95% confidence intervals using 1,000 bootstrap replications. Model selection was guided by Akaike Information Criterion (AIC) and Bayesian Information Criterion (BIC). Initially, conditional independence between diagnostic tests was assumed. If the model fit was inadequate, a model accounting for local dependence between tests was explored. Model goodness-of-fit was assessed using the bootstrap Pearson chi-square test, where a p-value greater than 0.05 indicated acceptable model fit.

A secondary analysis evaluated the agreement between PSTs and radiographic findings using Cohen’s kappa statistics with 95% confidence intervals. The percent agreement between the diagnostic methods was also calculated. Kappa values were interpreted according to standard criteria as poor, fair, moderate, substantial, or almost perfect agreement.

## Results

Table 2 presents the baseline demographic and clinical characteristics of the enrolled participants and their teeth. A total of 100 teeth were included in the analysis, with a mean patient age of 42.2 ± 11.8 years. There were 66 (66.0%) male participants and 34 (34.0%) female participants. Perio-origin lesions demonstrated comparatively greater PPD, while multirooted teeth were more frequently associated with true combined lesions, with 16 of 24 lesions (66.7%) occurring in multirooted teeth.

**Table 2:** Baseline demographic and clinical characteristics of the study participants and teeth. Data presented as mean ± standard deviation and frequency (n) with percentage (%). Descriptive statistics were used for analysis. Percentages were calculated within lesion categories. SD: standard deviation. PPD: probing pocket depth.

Characteristic		Overall (N = 100 teeth)	Endo-origin (n = 42)	Perio-origin (n = 34)	True combined (n = 24)
Patient demographics	Age (years), mean ± SD	42.2 ± 11.8	44.6 ± 12.1	42.5 ± 12.4	40.6 ± 10.5
	Female, n (%)	34 (34.0%)	17 (40.5%)	11 (32.4%)	6 (25.0%)
	Male, n (%)	66 (66.0%)	25 (59.5%)	23 (67.6%)	18 (75.0%)
Tooth and lesion characteristics	PPD (mm), mean ± SD	5.35 ± 1.67	5.15 ± 1.62	5.65 ± 1.35	5.42 ± 1.68
	Tooth mobility grade II–III, n (%)	30 (30.0%)	10 (23.8%)	12 (35.3%)	8 (33.3%)
	Multi-rooted tooth, n (%)	42 (42.0%)	12 (28.6%)	14 (41.2%)	16 (66.7%)

Table 3 shows the cross-tabulation between the PST outcomes and radiographic classification. Among endo-origin lesions, most teeth showed non-vital pulp responses, observed in 32 (76.2%) cases, whereas only 2 (4.8%) teeth demonstrated normal sensibility responses. In contrast, normal PST responses were predominantly associated with perio-origin lesions, as observed in 18 teeth (52.9%). No hyperresponsive responses were recorded in any lesion category. True combined lesions exhibited mixed responses, with non-vital findings identified in 12 teeth (50.0%). The cross-tabulation findings indicate general concordance between PST outcomes and radiographic classifications, although the results should be interpreted cautiously given the potential for diagnostic misclassification.

**Table 3:** Cross-tabulation of PST outcomes according to radiographic classification. Data presented as frequency (n) and percentage (%). Percentages were calculated column-wise within radiographic categories. Cross-tabulation analysis was performed. Radiographic classification was compared with PST outcomes. PST: pulp sensibility test.

Pulp vitality test outcome	Endo-origin (n = 42)	Perio-origin (n = 34)	True combined (n = 24)	Total (N = 100)
Normal (vital)	2 (4.8%)	18 (52.9%)	5 (20.8%)	25
Hyperresponsive	0 (0.0%)	0 (0.0%)	0 (0.0%)	0
Hyporesponsive	8 (19.0%)	10 (29.4%)	7 (29.2%)	25
Negative	32 (76.2%)	6 (17.7%)	12 (50.0%)	50
Total	42 (42.0%)	34 (34.0%)	24 (24.0%)	100

Table 4 presents the LCA findings and diagnostic accuracy estimates. The model demonstrated an acceptable fit with a bootstrap Pearson chi-square value of 2.14 and a non-significant goodness-of-fit result (p = 0.34). The estimated prevalence of true endodontic/non-vital states was 62% (95% CI, 52-72%; p < 0.001). PSTs demonstrated higher estimated sensitivity (86%; 95% CI: 79-93%) than radiographic evaluation (73%; 95% CI: 64-82%), whereas radiographic findings demonstrated higher estimated specificity (85%; 95% CI: 76-94%) than PSTs (78%; 95% CI: 68-88%).

**Table 4:** LCA model fit and diagnostic accuracy estimates of PSTs and radiographic findings. LCA was performed for diagnostic accuracy estimation, and the bootstrap method with 1,000 replications was used to estimate CI. The bootstrap Pearson chi-square was 2.14 (p = 0.34), indicating acceptable model fit; AIC = 245.6, BIC = 258.3. Sensitivity and specificity estimates were derived from LCA and were referenced to a latent disease construct representing endodontic/non-vital versus periodontal/vital states rather than an external clinical gold standard. Reported p-values represent comparisons between the diagnostic accuracy estimates of pulp sensibility testing and radiographic assessment and do not represent significance testing of individual sensitivity or specificity estimates. *p < 0.05 considered statistically significant. LCA: latent class analysis; PST: pulp sensibility test; CI: confidence interval; AIC: Akaike Information Criterion; BIC: Bayesian Information Criterion.

Parameter	Estimate	95% CI	Between-test comparison p-value
Prevalence of true endo/non-vital state	0.62	0.52–0.72	<0.001*
PST sensitivity	0.86	0.79–0.93	0.018*
Radiograph sensitivity	0.73	0.64–0.82	
PST specificity	0.78	0.68–0.88	0.092
Radiograph specificity	0.85	0.76–0.94	

Table 5 summarizes the agreement between PSTs and radiographic findings. The overall agreement between the two diagnostic methods was 54.0%, with a Cohen’s kappa value of 0.33, indicating a fair agreement (p < 0.001). After binary recoding of the diagnostic categories, the agreement improved to 71.0%, with a kappa value of 0.43 representing moderate agreement (p < 0.001). These findings suggest that the two diagnostic methods are related but not interchangeable.

**Table 5:** Agreement between PSTs and radiographic findings. CIs calculated at 95%. For binary agreement analysis, endo-origin lesions and non-vital PST outcomes were classified as endodontic/non-vital, whereas perio-origin lesions and vital PST outcomes were classified as periodontal/vital. True combined lesions and reduced vitality responses were assigned according to their predominant diagnostic classification to maintain consistency with the binary latent disease structure used in the LCA. PST: pulp sensibility test; CI: confidence interval; LCA: latent class analysis.

Agreement metric	Value	95% CI
Overall percent agreement (3 × 3 classification)	54.0%	44.2% – 63.8%
Cohen’s kappa (3 × 3 classification)	0.33	0.21–0.45
Binary recoded agreement		
Percent agreement	71.0%	62.1%–79.9%
Cohen’s kappa	0.43	0.29–0.57

Table 6 presents the LCA-derived predictive values and sensitivity analysis results. Pulp sensibility testing demonstrated a positive predictive value (PPV) of 86% and a negative predictive value (NPV) of 79%, whereas radiographic examination demonstrated a PPV of 88% and a comparatively low NPV of 66%. Sensitivity analysis allowing for local dependence between tests demonstrated no statistically significant changes in sensitivity, specificity, or prevalence estimates (p > 0.05), confirming the robustness and stability of the primary LCA model.

**Table 6:** Predictive values and sensitivity analysis of LCA models. Sensitivity analysis was performed using LCA with local dependence adjustment. Wald z-tests were used to compare individual parameter estimates between models, and the corresponding z-values and p-values are reported. Overall model fit was compared using the likelihood-ratio chi-square test (χ² = 0.92, p = 0.34), which demonstrated no significant improvement in model fit with the local dependence model. A p-value <0.05 was considered statistically significant. Δ = Local dependence model estimate − Independence model estimate. LCA: latent class analysis; PST: pulp sensibility test.

Parameter	Independence model estimate	Independence model 95% CI	Local dependence model estimate	Local dependence model 95% CI	Δ	Wald z-value	p-value
PST sensitivity	0.86	0.79–0.93	0.84	0.77–0.91	−0.02	0.28	0.78
PST specificity	0.78	0.68–0.88	0.80	0.70–0.90	+0.02	0.47	0.64
Radiograph sensitivity	0.73	0.64–0.82	0.75	0.66–0.84	+0.02	0.59	0.55
Radiograph specificity	0.85	0.76–0.94	0.83	0.74–0.92	−0.02	0.71	0.48
Prevalence	0.62	0.52–0.72	0.60	0.50–0.70	−0.02	0.39	0.70

## Discussion

This study evaluated and compared the diagnostic accuracy of PSTs and radiographic findings in the diagnosis of endo-perio lesions using LCA. Accurate differentiation between endodontic and periodontal involvement is clinically important because treatment planning, prognosis, and long-term tooth survival depend on correct diagnosis. The findings of this study demonstrated that PSTs showed significantly higher sensitivity than radiographic examination for identifying true endodontic/non-vital lesions, whereas radiographic evaluation demonstrated slightly greater specificity. These findings emphasize the complementary role of clinical sensibility testing and radiographic assessment in the diagnosis of endo-perio lesions.

In this study, most teeth classified radiographically as having endo-origin lesions demonstrated non-vital pulp responses, whereas those with perio-origin lesions predominantly showed normal pulp responses. This observation is biologically plausible because pulpal necrosis is commonly associated with periapical inflammatory destruction, whereas periodontal lesions without pulpal involvement may maintain pulpal vitality for prolonged periods [11]. The anatomical communication pathways between the pulp and periodontium, such as lateral canals and dentinal tubules, facilitate microbial spread and contribute to overlapping disease presentations [11]. Similar observations were reported by Simon et al. [12], who emphasized that the origin of the lesion strongly influences the pulpal status and treatment outcome. Likewise, Rotstein and Simon [13] highlighted that endo-origin lesions frequently present with non-vital pulps, whereas primary perio-origin lesions generally retain vitality, unless secondary pulpal involvement occurs.

The LCA in this study demonstrated that PSTs had a higher sensitivity than radiographic findings for detecting endodontic/non-vital conditions. This may be attributed to the fact that PSTs assess the functional sensory response of the pulp before extensive structural radiographic changes become apparent. Conventional diagnostic approaches, including radiographic assessment, may not always accurately reflect the underlying biological status of the pulp, particularly during the early stages of disease progression [14]. These findings are consistent with previous reports suggesting that cold testing is a reliable PST for assessing pulpal status. Balevi [9] reported that cold pulp testing demonstrated favorable diagnostic accuracy compared with several other sensibility testing methods. Similarly, Jespersen et al. [15] observed that thermal testing provides high sensitivity for identifying pulpal necrosis when standardized techniques are employed. Nevertheless, PSTs are not without limitations. Conditions such as pulpal calcification, a history of dental trauma, and extensive restorations may alter neural responses and lead to false-positive or false-negative results. Although standardized testing protocols and eligibility criteria were used in this study, the potential influence of these factors on diagnostic performance cannot be completely excluded.

Although radiographic evaluation demonstrated a comparatively lower sensitivity, it showed a higher specificity in this study. This suggests that characteristic radiographic findings, such as periapical radiolucency, furcation involvement, and vertical bone loss, may be more useful for confirming the presence of the disease than for excluding it. Radiographs provide valuable structural information regarding the extent and pattern of hard tissue destruction; however, their interpretation may be limited by two-dimensional image acquisition and anatomical superimposition [16]. Previous studies by Patel et al. [17] and Estrela et al. [18] similarly demonstrated that radiographic interpretation alone may underestimate the true extent of endodontic lesions, particularly in early stages. The use of CBCT in selected cases improves visualization and diagnostic confidence, although radiation exposure and cost remain limiting factors.

The agreement analysis in this study revealed a fair-to-moderate agreement between PSTs and radiographic findings. This indicates that, although both methods are diagnostically related, they assess different biological aspects of disease progression. PSTs primarily evaluate the neural response within the pulp tissue, whereas radiographs assess structural changes in the surrounding hard tissues [5]. Therefore, discordant findings may occur, particularly in chronic lesions, calcified teeth, and cases of extensive periodontal destruction. A similar moderate agreement between clinical sensibility testing and radiographic interpretation has been documented in a systematic review by Mejàre et al. [19], who reported that combining clinical examination with radiographic findings significantly improves diagnostic accuracy compared with using either method alone.

One of the major strengths of this study was the use of LCA. In endodontic and periodontal diagnoses, a true gold standard is often unavailable, because histological confirmation is impractical in routine clinical settings. Latent class analysis provides a statistically robust approach for estimating diagnostic accuracy, without requiring a perfect reference standard. The stability of the sensitivity analysis results further supports the reliability of our findings. To our knowledge, there is limited literature evaluating diagnostic methods for endo-perio lesions using advanced statistical approaches such as latent class analysis, highlighting the clinical and methodological relevance of this study.

The clinical implications of these findings are significant. The higher sensitivity of pulp sensibility testing suggests that PSTs should remain a primary diagnostic tool for identifying endodontic involvement in endo-perio lesions. However, radiographic examination remains indispensable for evaluating the extent and pattern of bone destruction and for confirming structural changes associated with disease progression. Therefore, clinicians should avoid relying solely on either pulp sensibility testing or radiographic findings and instead use a combined diagnostic approach to improve accuracy and treatment planning. Early and accurate diagnosis may facilitate appropriate treatment selection and clinical decision-making for endo-perio lesions.

Despite its strengths, this study had several limitations. The study was conducted at a single center with a relatively limited sample size, which may affect the generalizability of the findings. Although LCA reduces dependence on a gold standard, diagnostic estimates remain model-dependent. The LCA employed a binary latent disease structure representing predominantly endodontic/non-vital and periodontal/vital disease states. Although this approach was selected to address the primary diagnostic comparison and maintain model stability with the available sample size, it may not fully capture the biological complexity of true combined endo-perio lesions. Furthermore, the latent class model assumed conditional independence between PST outcomes and radiographic findings; therefore, the possibility of residual dependence influencing parameter estimates cannot be completely excluded. Future investigations using larger cohorts and multiclass latent class models may provide a more nuanced characterization of these lesions. Additionally, patient-reported responses during sensibility testing may introduce subjective variability. CBCT imaging was performed only when conventional periapical radiographs provided insufficient visualization; therefore, the radiographic findings represent a combined radiographic assessment approach, and some degree of verification bias cannot be excluded. Future multicenter studies with larger sample sizes and the incorporation of advanced pulp vitality assessment methods, such as laser Doppler flowmetry and pulse oximetry, may provide further insight into the diagnosis of endo-perio lesions.

## Conclusions

Within the limitations of this study, PSTs demonstrated higher sensitivity than radiographic assessment for identifying endodontic/non-vital involvement in endo-perio lesions, whereas radiographic findings showed comparatively greater specificity. The findings suggest that the two diagnostic modalities provide complementary information and may be most effective when interpreted together. A combined approach incorporating pulp sensibility testing and radiographic assessment may improve diagnostic confidence and support clinical decision-making in the evaluation of endo-perio lesions. Given the cross-sectional design and the model-dependent nature of the diagnostic estimates, the results should be interpreted with caution and validated in larger prospective studies.
